# Deciduous Trees and the Application of Universal DNA Barcodes: A Case Study on the Circumpolar *Fraxinus*

**DOI:** 10.1371/journal.pone.0034089

**Published:** 2012-03-27

**Authors:** Mariangela Arca, Damien Daniel Hinsinger, Corinne Cruaud, Annie Tillier, Jean Bousquet, Nathalie Frascaria-Lacoste

**Affiliations:** 1 Université Paris Sud, UMR 8079, Orsay, France; 2 Centre national de la recherche scientifique, UMR 8079, Orsay, France; 3 AgroParisTech, UMR 8079, Orsay, France; 4 Chaire de recherche du Canada en génomique forestière et environnementale, Centre d'étude de la forêt, Université Laval, Québec, Québec, Canada; 5 Génoscope, Centre national de séquençage, Evry, France; 6 Département systématique et évolution and Service de systématique moléculaire, Muséum national d'histoire naturelle, Paris, France; National Institute on Aging, United States of America

## Abstract

The utility of DNA barcoding for identifying representative specimens of the circumpolar tree genus *Fraxinus* (56 species) was investigated. We examined the genetic variability of several loci suggested in chloroplast DNA barcode protocols such as *matK*, *rpoB*, *rpoC1* and *trnH-psbA* in a large worldwide sample of *Fraxinus* species. The chloroplast intergenic spacer *rpl32-trnL* was further assessed in search for a potentially variable and useful locus. The results of the study suggest that the proposed cpDNA loci, alone or in combination, cannot fully discriminate among species because of the generally low rates of substitution in the chloroplast genome of *Fraxinus*. The intergenic spacer *trnH-psbA* was the best performing locus, but genetic distance-based discrimination was moderately successful and only resulted in the separation of the samples at the subgenus level. Use of the BLAST approach was better than the neighbor-joining tree reconstruction method with pairwise Kimura's two-parameter rates of substitution, but allowed for the correct identification of only less than half of the species sampled. Such rates are substantially lower than the success rate required for a standardised barcoding approach. Consequently, the current cpDNA barcodes are inadequate to fully discriminate *Fraxinus* species. Given that a low rate of substitution is common among the plastid genomes of trees, the use of the plant cpDNA “universal” barcode may not be suitable for the safe identification of tree species below a generic or sectional level. Supplementary barcoding loci of the nuclear genome and alternative solutions are proposed and discussed.

## Introduction

Over the past decade, several protocols for identifying species from short orthologous DNA sequences, known as DNA barcodes, have been proposed. They have been promoted as useful for the rapid identification and discovery of species and applied to biodiversity studies [1]. Created in 2004, the “Consortium for the Barcode of Life” (CBOL) proposed that this approach should be used to create a global DNA barcode database of biodiversity using standard short genomic regions that are present universally among species, or BOLD (Barcode Of Life Data systems, [2]).

Barcoding relying on the mitochondrial gene coding for cytochrome c oxidase (*cox1* or *co1*) has been used successfully to identify species in various animal taxa, including birds [3], [4], butterflies [5], [6], [7], bats [8], and fish [9]. However, *cox1* and other mitochondrial genes are not suitable as barcodes for plants because of their very low rates of substitution in plants [10], [11]. Moreover, frequent hybridisation, polyploidy, and apomixis in plants make the identification of an ideal barcode locus more difficult than in animals [12].

The circumpolar tree genus *Fraxinus* (Oleaceae) comprises about 45 tree species mainly distributed in the temperate but also subtropical regions of the northern hemisphere [13], [14]. As such, they are well representative of temperate and boreal trees in terms of life history and population genetics attributes [15]. The monophyly of the genus in the tribe Oleeae has been confirmed [16] and six sections (*Dipetaleae*, *Fraxinus*, *Melioides*, *Ornus*, *Pauciflorae* and *Sciadanthus*) have been delineated on the basis of molecular (reciprocal monophyly) and morphological characters (flowers and samara morphology) [13] (Table 1). The species found in the different sections usually form cohesive continental groups (North America for the sections *Dipetaleae*, *Melioides* and *Pauciflorae*; Eurasia for the sections *Fraxinus*, *Ornus* and *Sciadanthus*). Many ash species have commercial uses for the quality of their wood or for their chemical components [17]. Moreover, some species are threatened or endangered at the international level (*F. sogdiana* and *F. hondurensis*, listed on the Red List of the IUCN), national (*F. mandshurica* in China) or regional scale (*F. profunda* in Michigan, New Jersey and Pennsylvania, *F. quadrangulata* in Iowa and Wisconsin, *F. parryi* in California). Despite the fact that a majority of species could be easily identified in the field, the systematic relationships among sections and groups in the genus are not entirely set [18], [19]. Some closely related species have also been shown to hybridize in sympatric areas, complicating the morphological identification of individual trees (e.g [20]). The use of exotic ashes in certain countries (e.g. Reunion island, Ireland) has also revealed emerging problems related to the purity of commercial seeds used for reforestation [21]. These factors make the development of reliable identification tools urgent in the genus, especially when access to reliable morphological information is absent or limited.

**Table 1:** Classification of the genus *Fraxinus* and geographical distributions of species.

Section	Species	Synonyms used in this study	Distribution
*Ornus*	*ornus* L.		Mediterranean area, N Africa and SW Asia
	*apertisquamifera* Hara		Japan
	*bungeana* DC		China
	*floribunda* Wall.	*retusa* Champ. ex Benth.var. *henryana*	C & E Asia (from Afghanistan to Japan)
	*griffithii* G. B. Clarke		E Asia (from NE India to Japan and Indonesia)
	*lanuginosa* Koidz. var. *lanuginosa* and var. *serrata* (Nakai) Hara		Japan
	*malacophylla* Hemsl.		China, Thailand
	*paxiana* Lingelsh.	*sikkimensis* (Lingelsh.) Handel-Mazzetti	Himalayas, China
	*raibocarpa* Regel		C Asia (Turkestan mountains, Iran, Pakistan, Afghanistan)
	*sieboldiana* Blume	*mariesii* Hook. f.	China, Japan, Korea
	*trifoliolata* W. W. Smith		China
	*baroniana* Diels		China
	*chinensis* Roxb.		China, Japan, Korea, Vietnam
	*longicuspis* Sieb. & Zucc.		Japan
	*micrantha* Lingelsh.		C Asia (Punjab to Nepal, Himalayas)
*Dipetalae*	*anomala* Torr. ex S. Wats.		SW USA, N Mexico
	*dipetala* Hook. et Arn.	*trifoliata* (Torr.) Lewis & Epling	SW USA, N Mexico (Baja California)
	*quadrangulata* Michx.		E & C USA, C Canada
*Fraxinus*	*angustifolia* Vahl. ssp. *angustifolia*	*monophylla* Desf.	SW Europe
	*angustifolia* Vahl. ssp. *oxycarpa* (M.Bieb. ex Willd.) Franco & Rocha Afonso	*oxycarpa* Willd., *pallisiae* A. J. Willmott, *obliqua* Tausch	SE Europe
	*angustifolia* Vahl. ssp. *syriaca* (Boiss.) Yalt.	*potamophila* Herder, *holotricha* Koehne, *sogdiana* Bunge, s*yriaca* Boiss.	W & E Asia (Turkey to Pakistan and Russia) and Algeria
	*excelsior* L.	*turkestanica* Carrière	C & N Europe
	*mandshurica* Rupr.		E Asia (China, Japan, Korea, E Russia)
	*nigra* Marsh.		E USA, E Canada
	*platypoda* Oliv.		E Asia
*Melioides*	*americana* L.	*biltmoreana* Beadle	E USA, E Canada
	*berlandieriana* A. DC.		SW USA, NE Mexico
	*caroliniana* Mill.		SE USA
	*latifolia* Benth.		W USA
	*papillosa* Lingelsh.		SW USA (SE Arizona, SW New Mexico, Texas), Mexico (W Chihuahua, NE Sonora)
	*pennsylvanica* Marsh.	*richardii* Bosc	C & E USA, Canada
	*profunda* (Bush) Bush	*tomentosa* Michx. f.	E USA
	*texensis* A. Gray		S USA (Texas)
	*uhdei* (Wenzig) Lingelsh.		Guatemala, Honduras, Mexico, USA (Hawaii, Puerto Rico)
	*velutina* Torr		SW USA, N Mexico
*incertae sedis*	*cuspidata* Torr.		SW USA, Mexico
	*chiisanensis* Nakai		Korea
	*spaethiana* Lingelsh.		Japan

Asterisk indicates synonyms as tagged in the arboreta. Adapted from Wallander [13].

A variety of loci widely used in phylogenetic studies have been suggested as DNA barcodes for plants, as recently reviewed [22]. These include chloroplast genes such as *rbcL* [23], *ndhF* [24], and *matK* [25], and non-coding spacers such as the *trnL* intron [26], [27], *trnH-psbA* [28] and *trnT–trnL* [29] in the chloroplast genome (see [22]). However, none of these regions presents a sufficiently high rate of substitution to allow plant species to be distinguished using a single locus barcode. Some nuclear loci have been proposed too [30], such as the ribosomal nuclear intergenic transcribed spacer (ITS) [31], [32], [33], [34], [35], or the external transcribed spacer (ETS) [36]. Both loci exhibit generally a much higher level of variation than chloroplast genes [37], [38], high level of concerted evolution [39], and more or less rapid fixation of new variants [40]. However, the presence of paralogous variation in many taxonomic groups has prevented until now the use of nuclear ribosomal spacers as barcode at a large scale. Therefore, the necessity for a more complex multilocus approach has been suggested [25], [31], [41].

A standardised plant barcode has been proposed by Chase et al. [42], then by CBOL [32]. Both of these barcodes rely on a cpDNA multilocus approach, and the loci used have been extensively described (see [22] for a review). The CBOL approach combines two cpDNA regions, *matK* and *rbcL*. These two regions present good features such as easy routine amplification and sequencing using universal primers, especially for *rbcL* [22]. Because *matK* usually shows two- to threefold higher substitution rates than *rbcL* [43], [44], [45], [46], it is usually used for the discrimination of congeneric taxa. The substitution rates of *rbcL* appear especially low in perennial and woody angiosperm taxa [47], [48], which make it more suited for studies at a variety of higher taxonomic levels, from intergeneric to subclass [49], [50]. For this reason, its inclusion in the CBOL barcode protocol is usually for anchoring taxa at the generic level [32]. While ashes can be easily discriminated from other Oleaceae genera using morphological traits alone [51], *rbcL* conforms to the general pattern in that it presents little variation for discriminating ash taxa. Indeed, a GenBank survey of *rbcL* sequences made in preparation to this study indicated that the two sections *Ornus* and *Fraxinus* exhibited only one substitution (0,2%) among the five sequences available (*F. chinensis* DQ673301, *F. ornus* FJ862057 for the section *Ornus*, *F. excelsior* FJ395592 and FJ862056 and *F. angustifolia* FJ862055 for the section *Fraxinus*). Moreover, this unique substitution was an apomorphy, thus presenting little value as a diagnostic marker for the sectional level. Due to such low levels of interspecific variation, *rbcL* cannot be considered as a potential candidate for DNA barcode in ashes, except for eventually assigning an unknown sample to the genus.

In the present study, we focused on testing the standardised barcode of Chase et al. [42] because in addition to the reputedly variable *matK* locus already suggested by the CBOL, it proposes additional cpDNA loci for potentially useful discrimination among congeneric taxa. The barcode protocol by Chase et al. [42] is based on two different combinations of three separate plastid regions: option 1 comprises the three genes *rpoC1*, *rpoB*, and *matK,* whereas option 2 relies on an intergenic spacer region, *trnH–psbA*, in addition to *rpoC1* and *matK*. The non-coding plastid region *trnH–psbA* was first proposed by Kress et al. [31], who compared nine candidate barcode cpDNA loci, which included coding and non-coding regions. It was shown that the level of discrimination increased when a non-coding spacer was paired with one of three coding loci tested. Moreover, it has been shown that *trnH-psbA* exhibits higher species discrimination power than *rbcl* and *matK* combined in some tree genera [22].

Despite the increasing number of reports on the effectiveness of these candidate plant barcode loci, most of them concerned herbaceous or shrub taxa [24], [29], [52], [53], [54], [55], [56], [57], with still few studies about tree and other long-living plant taxa [58], [59], [60]. Testing trees is important as they have been shown to harbor generally large population sizes, lower substitution rates per unit of time and lower diversification rates than annual plant species (for a review, see [61]).

Our goal was to assess the efficacy of the two options of the standardised DNA barcode proposed by Chase et al. [42] for discriminating morphologically well-defined species of the genus *Fraxinus*, and test for this purpose an additional variable and potentially useful region of the chloroplast genome, the *rpl32-trnL* spacer [62]. To explore the utility of these loci, we further tested them in conjunction with two numerical methods, the Nearest Neighbour algorithm (through NJ trees) and the BLAST algorithm.

## Results

**Figure 1:**
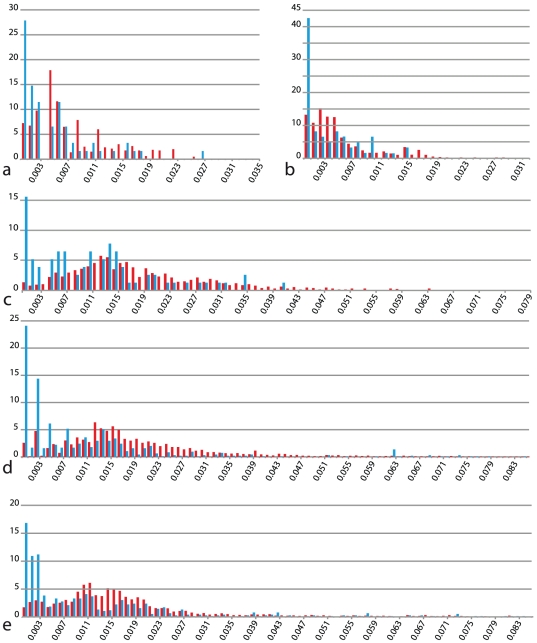
Intraspecific (blue) and interspecific (red) rates of substitution per 100 sites for each cpDNA region tested. X-axis is K2P substitution rate. Y-axis is relative frequency within each dataset. a, *matK* dataset; b, barcode option 1 (*rpoC1*, *rpoB* and *matK*); c, barcode option 2 (*rpoC1*, *matK* and *trnH-psbA*); d, *trnH-psbA*; e, *rpl32-trnL*.

Forty-two (80.8%), 44 (84.6%), 41 (78.8%), 226 (88.3%), and 202 (78.9%) samples from *Fraxinus* were amplified and sequenced successfully for *matK*, *rpoC1*, *rpob*, *trnH-psbA*, and *rpl32-trnL*, respectively (details in Table S1). K2P pairwise substitution rates calculated for each dataset showed very low sequence divergence values (Table 2) and the lack of the typical barcode gap, a trend that indicated a large overlap between intraspecific and interspecific pairwise distances (Fig. 1). The average difference considering the entire dataset was only 0.6%, ranging from 0.2 to 0.9% (Table 2).

**Table 2:** Sequence variation and discrimination power of the cpDNA barcode regions in *Fraxinus* spp.

	Number of species considered	Number of samples analysed	Number of nucleotide sites	Variable nucleotide sites (percent)	Diagnostic nucleotide sites^1^ (percent)	Minimum pairwise substitution rate per 100 sites	Maximum pairwise substitution rate per 100 sites	Mean intraspecific distance per 100 sites (min-max)	Mean interspecific distance per 100 sites (min-max)	Nb. of species identified by NJ tree	Nb. of species identified by Blast search
**Reduced dataset**											
Option 1 (*rpoC1*, *rpoB*, *matK*)	23	52	1704	95 (5.6)	24 (1.4)	0	3.1	0.3 (0–1.4)	0.5 (0–3.1)	1	-
Option 2 (*rpoC1*, *matK*, *trnH-psbA*)	23	52	1909	231 (12.1)	72 (3.8)	0	8.4	1.1 (0–4.1)	1.9 (0–8.8)	2	-
*matK*	23	52	721	61 (8.5)	19 (2.6)	0	3.8	0.5 (0–2.7)	0.8 (0–3.5)	2	-
**Expanded dataset**											
*trnH–psbA*	56	226	698	203 (29.1)	107 (15.3)	0	12.5	1.0 (0–8.8)	2.0 (0–13.0)	8	9
*rpl32–trnL*	56	202	1023	451 (44.1)	193 (18.9)	0	16.5	1.2 (0–15.5)	1.9 (0–16.0)	3	-

1 Equivalent to phylogenetically informative sites.

Intraspecific and interspecific distances calculated using the pairwise Kimura 2-parameter (K2P) substitution rates, and NJ tree based on K2P substitution rates.

### Reduced dataset

Barcode option 1 (*matK*, *rpoC1*, *rpob*) was tested with 27 samples sequenced for the three loci and 48 samples sequenced for at least two loci, and barcode option 2 (*matK*, *rpoC1*, *trnH-psbA*) was tested for 23 and 48 samples sequenced for three and two loci, respectively. The loci *rpoC1*, *rpoB* and *matK* resulted in a single amplicon for almost all samples. In a population sample for each of *F. excelsior* and *F. angustifolia* (25 individuals per species), the two species were polyphyletic and could not be differentiated because no diagnostic or synapomorphic polymorphisms were detected (results not shown). For this dataset, only one indel was found in each region after aligning the sequences: a 3-bp insertion in *rpoC1* in one individual of *F. quadrangulata*, a 9-bp deletion in *matK* of *F. mariesii*, and a 12-bp insertion in *rpoB* for all *Fraxinus* taxa, but not in the outgroup *Jasminum nudiflorum*.

The alignment of the chloroplast *rpoC1* and *rpoB* gene sequences was straightforward and revealed a small number of variable sites for each of the barcode options 1 or 2 (Table 2). Sequence diversity was relatively low: the proportion of variable sites was 3.8% in *rpoC1*, 3.0% in *rpoB*, and 3.8% in *matK*. *MatK* and barcode option 1, which implicates *matK* in combination with *rpoC1* and *rpoB*, appeared to be the most afflicted by the lack of clear delineation between intraspecific and interspecific levels of sequence polymorphism. The differences between the maximum pairwise intraspecific and interspecific distances were 0.3% for *matK* and 0.2% for the barcode option 1 (Table 2). *trnH-psbA* was the most variable marker of both options (see Expanded dataset).

**Figure 2:**
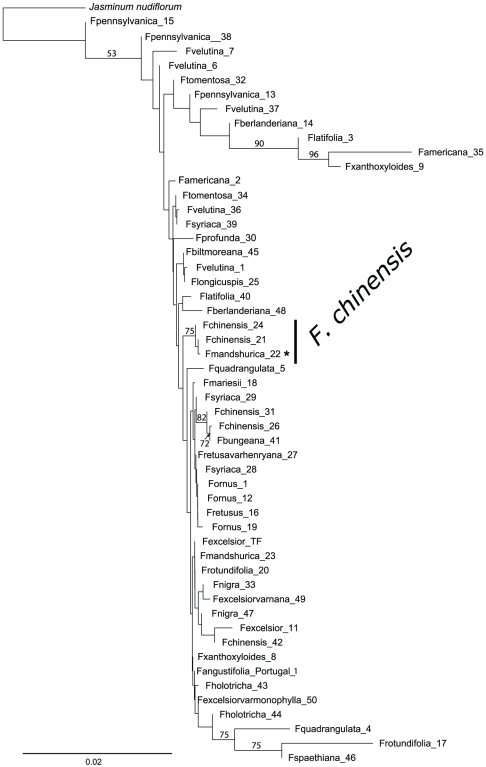
NJ tree of pairwise K2P substitution rates for the barcode option 1 (*rpoC1*, *rpoB* and *matK*) implicating the reduced dataset. Bootstrap values of 50% and above are shown on the branches. Species that were potentially well-delineated with these sequences are marked by a black vertical line. Individuals marked by asterisks were likely misidentified, and not considered in species delineations. The scale bar represents the substitution rate per 100 sites.

**Figure 3:**
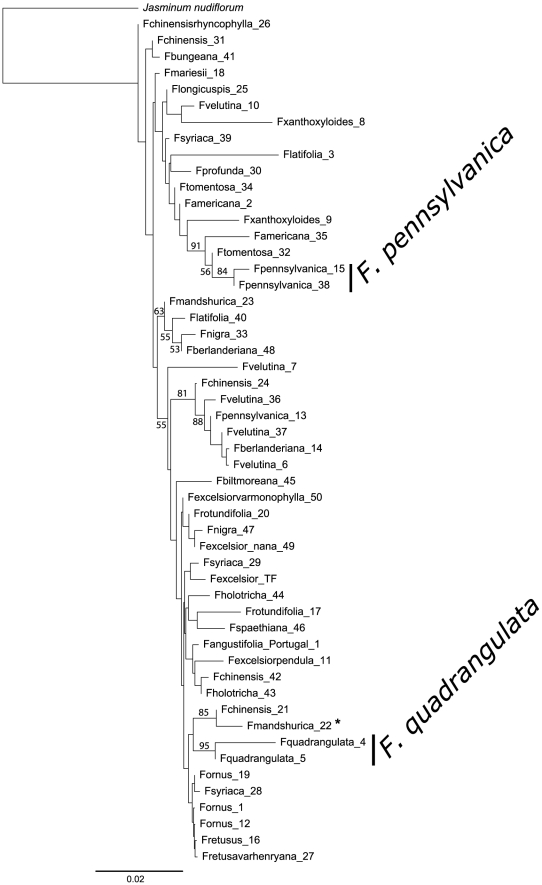
NJ tree of pairwise K2P substitution rates for the barcode option 2 (*rpoC1*, *matK* and *trnH-psbA*) implicating the reduced dataset. Bootstrap values of 50% and above are shown on the branches. Species that were potentially well-delineated with these sequences are marked by a black vertical line. Individuals marked by asterisks were likely misidentified, and not considered in species delineations. The scale bar represents the substitution rate per 100 sites.

The NJ tree of K2P substitution rates that resulted from the application of barcode option 1 to the reduced dataset showed only one interesting group, which consisted of the samples of *F. chinensis* and included a specimen of *F. mandshurica* (belonging to a different taxonomical section), which had probably been misidentified in the arboretum (Fig. 2). We found no other case of misidentification in our dataset. It should also be noted that this group did not include all samples from *F. chinensis*. The minimum NJ tree of K2P substitution rates that derived from barcode option 2 delineated only two monospecific groups: *F. quadrangulata* and *F. pennsylvanica* (Fig. 3). The former group included all specimens available for this species, but not the second one. Both NJ trees showed low bootstrap support for all nodes of interest, except *F. quadrangulata* for barcode option 2, which showed 95% support (Fig. 3).

### Expanded dataset

The alignment of *trnH-psbA* sequences was sometimes difficult or ambiguous due to numerous deletions. In the alignment of *trnH-psbA* (698 bp), 203 (29.1%) sites were variable but only 107 (15.3%) had some diagnostic value since they were shared by more than one individual per species. The *trnH–psbA* intergenic region contained 28 indels, with most of them being diagnostic for different sections of the genus. Notably, an insertion of 11 bp was noted in all *Fraxinus* sequences, which was absent in the outgroup *Jasminum nudiflorum*; a deletion of 196/197 bp was observed in some *F. velutina* specimens, and an insertion of 6 bp was noted in *F. quadrangulata*, which was shared with the outgroup *Jasminum nudiflorum*. Seventy-two Eurasian individuals from diverse species and sections (comprising 2 *F. angustifolia*, 8 *F. apertisquamifera*, 2 *F. bungeana*, 5 *F. chinensis*, 22 *F. lanuginosa*, 10 *F. longicuspis*, 1 *F. mandshurica* (Fmandshurica_212), 8 *F. ornus*, 4 *F. platypoda*, 8 *F. sieboldiana*, and 2 *F.* sp.) shared a 92-bp deletion, which suggests that the two specimens of *F. angustifolia* and the specimen of *F. mandshurica*, which was retrieved out of their section, had been misidentified, They might have been overlooked hybrids or introgressants, or have shared an ancestral polymorphism (see Materials and Methods).

**Figure 4:**
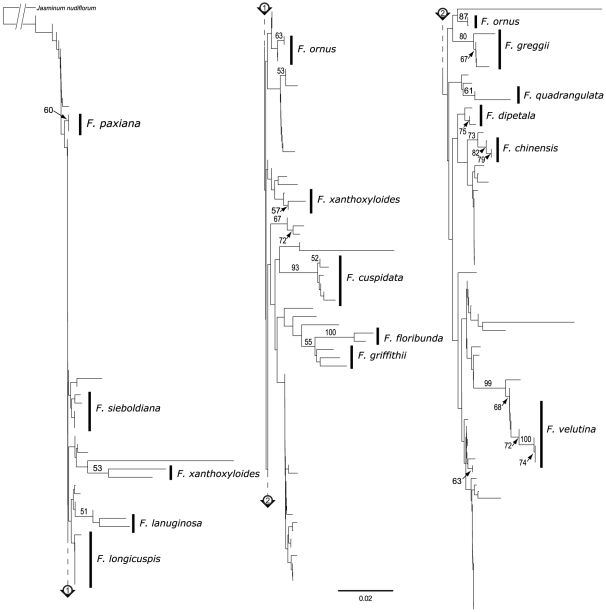
NJ tree of pairwise K2P substitution rates for the *trnH–psbA* dataset implicating the expanded dataset. Bootstrap values of 50% and above are shown on the branches. Species that were potentially well-delineated with these sequences are marked by a black vertical line. Individuals marked by asterisks were likely misidentified, and not considered in species delineations. The scale bar represents the substitution rate per 100 sites.

**Figure 5:**
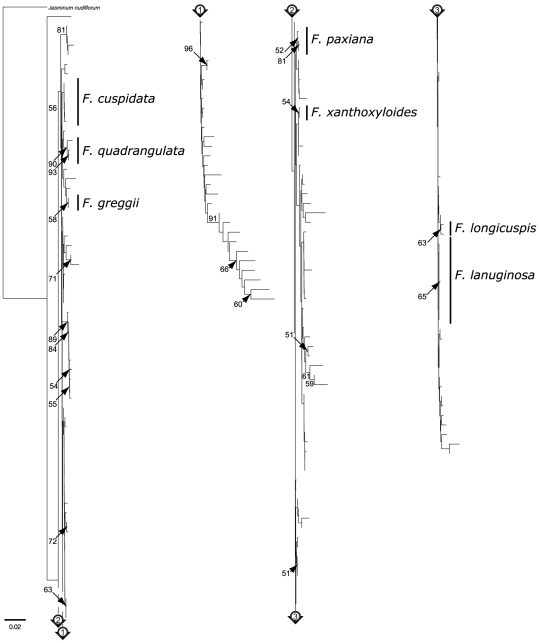
NJ tree of pairwise K2P substitution rates for the *rpl32–trnL* dataset implicating the expanded dataset. Bootstrap values of 50% and above are shown on the branches. Species that were potentially well-delineated with these sequences are marked by a black vertical line. The scale bar represents the substitution rate per 100 sites.

The minimum NJ tree of K2P substitution rates for the *trnH-psbA* dataset (Fig. 4) showed more encouraging results: 16 groups were monospecific and eight of them grouped more than 50% of the identified specimens of a given species (for *F. cuspidata*, *F. dipetala*, *F. floribunda*, *F. greggii*, *F. griffithii*, *F. paxiana*, *F. quadrangulata* and *F. velutina*). The bootstrap values for the groups of interest ranged from 51% to 100% and, in general, were high when all individuals of a given species were included in the group. Although the *rpl32–trnL* sequences showed more variation than *trnH-psbA* (Table 2), the NJ tree for *rpl32–trnL* (Fig. 5) showed a lower resolution than that for *trnH–psbA*, with three groups containing more than 50% of the individuals of a given species (for *F. greggii*, *F. paxiana* and *F. quadrangulata*) and with seven other monospecific groups. Notably, *F. quadrangulata* was the only monospecific group with a high bootstrap support (90%).

For the test case using the BLAST algorithm and based on the expanded dataset and the intergenic spacer sequences *trnH–psbA*, all specimens for nine species were correctly identified at the first hit (*F. anomala*, *F. griffithii*, *F. latifolia*, *F. ornus*, *F. paxiana*, *F. quadrangulata*, *F. sieboldiana*, *F. spaethiana* and *F. xanthoxyloides*, Table 2), and for 11 species at the second and third hits. Twelve species were correctly identified for more than 50% of the specimens considering only the first hit, and 17 species were correctly identified for more than 50% of the specimens, considering the first three hits (*F. angustifolia*, *F. anomala*, *F. chinensis*, *F. excelsior*, *F. griffithii*, *F. holotricha*, *F. latifolia*, *F. longicuspis*, *F. ornus*, *F. paxiana*, *F. platypoda*, *F. profunda*, *F. quadrangulata*, *F. sieboldiana*, *F. spaethiana*, *F. velutina* and *F. xanthoxyloides*). With respect to the recognition of the different sections of the genus, 83% of the *Dipetalae*, 44% of the *Fraxinus*, 89% of the *incertae sedis*, 22% of the *Melioides*, 58% of the *Ornus*, and 50% of the *Pauciflorae* individuals were correctly ascribed to their section, with an average of 51% correct section assignments, overall. In comparison, the more traditional approach, which relied on NJ analysis of K2P pairwise substitution rates based on the same locus and sample set, resulted in the correct discrimination of only seven species, based on the criterion that minimally more than 50% of the individuals of a given species be assigned to a unique species (Table 2) (see Methods).

## Discussion

Our results indicate that a substantial number of *Fraxinus* species could not be distinguished using either options of the standardised cpDNA plant barcode reported by Chase et al. [42] and using either methods of numerical analysis tested. The best case scenario was obtained with the BLAST approach applied to *trnH-psbA* intergenic sequences for the expanded dataset, where 32% of the species could be retrieved in the three first hits (all samples assigned to correct species). Our results showed that the tested DNA barcodes in their different configurations could only be used to perhaps confirm a previous morphological or molecular identification in the genus *Fraxinus*, even when using different methods of numerical analysis. Overall, the observed lack of discrimination power of the barcodes tested was more attributable to the low levels of nucleotide polymorphism of the diagnostic cpDNA regions investigated across *Fraxinus* taxa, rather than the numerical approach used to handle the sequence polymorphisms.

### Lack of variation of the tested barcodes in Fraxinus

Accurate identification using DNA barcodes requires that sufficient information is available at the interspecific level and between closely-related species so that most if not all species sampled show a clear diagnostic pattern. However, one could argue that species identification is not always a necessity, and that a piece of *Fraxinus* leaf or root tissue identified to a small set of possible species could be of enormous utility, and we agree with this view. Nonetheless, with the large set of cpDNA regions tested here, it appears that an ash sample could only be reliably assigned to the genus *Fraxinus*, and eventually to a section. Given that many species could belong to a section (for instance, 15 species in the section *Ornus*), that species from a same section could occur both in sympatry and allopatry, and show different types of use (traditional pharmacopeia, timber, *etc.*), and therefore different anthropogenic pressures, a sectional identification in ashes would be of little interest for practical use by non-taxonomists.

When considering the most variable cpDNA region of the barcode of Chase et al. [42], *trnH-psbA*, which has been tested here but not been retained in the most recent plant barcoding proposals [32], most polymorphisms were not fixed within species and 29% of the polymorphisms were shared between two *Fraxinus* species or more, particularly between taxa from the same geographic areas (e.g. Japan, Europe). This pattern suggests slow fixation rate related to incomplete lineage sorting or reticulate evolution [63], or recent divergence at several places in the genus, as documented in the *F. angustifolia* – *F. excelsior* species complex [15], [18], [20]. Thus, even if the *trnH–psbA* region was the least conserved and most informative among the cpDNA loci analysed, our results indicate that it would not represent a suitable locus for a standardised barcode approach for the non-specialist identification of plant material in the genus *Fraxinus*. It has also been shown that intraspecific inversions exist in some taxonomic groups, which would pose a further challenge to the use of *trnH-psbA* as a universal barcode [64]. Despite a promising level of polymorphism [62], the *rpl32-trnL* region also showed little variation in the genus *Fraxinus*. The *rpl32-trnL* NJ tree showed lower resolution than the tree resulting from the analysis of *trnH-psbA* sequences.

### Methodological considerations

The results derived from the analysis of *trnH–psbA* sequences for the expanded dataset indicate that the BLAST approach was slightly more powerful at distinguishing species than the use of substitution rates matrices and distance-based tree construction methods such as NJ. This is probably because distance-based methods combine all sites in each sequence in a single index, whereas the BLAST algorithm uses local comparisons, which are more sensitive to small differences. In our study, the BLAST algorithm outperformed the distance-based approach (NJ with K2P substitution rates) when relying on the most variable region, *trnH–psbA*. Although *trnH-psbA* was the most variable region tested with the two approaches, even the use of BLAST did not result in clear sample identification for most species. Several studies [53], [56], [65] recently proposed that different methods of analysis, such as graphical representation (multidimensional analysis), could be more effective than the distance-based NJ method, as recommended for animals [1]. However, these studies handled datasets with very low average sequence divergence between species (0.5% divergence in[56], 0.2% in [65]), had no bootstrap support indicated for the monospecific groups delineated [56], or had no tree-based representation of the results obtained [53], [65]. The question of a most suitable method for the delineation of groups or species including which phylogenetic method would be more adequate has been debated extensively over the past 20 years [66], [67], [68], [69].

### Finding a cpDNA barcode for Fraxinus

Our results indicate that a few highly probable morphological misidentifications (2 trees out of a total of 253) occurred in the herbaria and arboreta specimens sampled, despite the great care taken to validate all specimens *a priori* using morphology. An empirical study in the genus *Inga* [70], based on a field morphological identification and molecular fingerprinting, reported an error rate around 7% in morphological identification. The present rate of misidentification was low and did not affect the general findings of the study where too little sequence variation was observed for the proposed barcodes and cpDNA regions analysed to clearly discriminate ash species. Previous surveys of cpDNA polymorphisms were conducted for some species of the genus *Fraxinus*, confirming the maternal inheritance of cpDNA [71], and showing the lack of interspecific variation between four species from sections *Fraxinus* and *Melioides* for the chloroplast intron *trnL* and intergenic spacer *trnL-trnF* [72]. It has also been possible to discriminate *F. excelsior* from *F. oxyphylla* (presently known as *F. angustifolia*) in some mixed samples of common ash using a cpDNA simple sequence repeat (SSR) but, unfortunately, this maternal marker was less effective in hybrid zones involving these species [18]. Overall, ash species appear to show low levels of overall variation in cpDNA sequences, especially fixed interspecific differences. Moreover, it has been shown that trees and other perennial plants might have lower substitution rates per year than that of annual plants for chloroplast loci [48], [61]. These differences could be related to reduced mutation rate [61] or longer generations, larger population sizes, and reduced fixation rates in tree species [48]. Slow fixation rates could results in the polyphyly observed in our data and the previous phylogenies [13], [73], likely explained either by incomplete lineage sorting or by reticulation. The multiple instances of haplotype sharing noted between some of the ash species may indicate that these species are relatively recent on the geological time scale, with weak reproductive isolation. Indeed, natural hybridization has been reported between several ash species (e.g. [18], [20]), and it has been suspected between others species as well [74], [75]. Such reticulate evolution has been shown in Oleaceae (e.g. [76], [77]) and many other species [78], sometimes at a large scale in tree genera [79], [80], and it could surely account for part of the shared polymorphisms observed, at least between closely related species. Other factors such as incomplete lineage sorting, even between phylogenetically distant species [63], [81], could also prevent the recognition of species through DNA barcode in the genus *Fraxinus*. Indeed, the reproductive biology and apparent large population sizes characterizing ash species. may retard the fixation of ancestral polymorphisms within species [15]. Overall, *Fraxinus* combined many features (long-lived organisms, large population sizes, frequent hybridisation, species morphologically too narrowly defined) known to lower the success in species identification in barcoding studies [22].

### Barcoding in other tree taxa

Few barcode analyses at the species level have been reported in trees or long-living perennials, but some general conclusions can be made from the published data that used several cpDNA regions or regions of the nuclear genome. In the Oleaceae, only the nuclear ribosomal internal transcribed spacer (nITS) and the cpDNA *trnH-psbA* intergenic region harboured enough nucleotide polymorphisms to delineate and identify satisfactorily species in the genus *Ligustrum*, while *rbcL* and *matK* had poor discrimination [82]. Other case studies involving perennial genera generally resulted in mixed or negative results. For example, among gymnosperms, cycadales showed contrasting results, depending on the genus analysed [52]. Good species discrimination was obtained in some genera (*Mycrocycas*, *Strangeria*, *Lepidozamia*) using seven chloroplast loci whereas poor discrimination was obtained between closely-related species in *Encephalartos* [52] and in *Araucaria* [41]. Despite relying on many chloroplast loci, including standard ones, the cpDNA regions tested did not show sufficient variation to provide unique polymorphisms identifying single species, in addition to amplification problems [52]. Among basal angiosperms, Myristicaceae appeared to be more suited for DNA barcoding than gymnosperms [83], although the authors acknowledge “that many of the plastid regions suggested for plant barcoding will not differentiate species in *Compsoneura*”. They found that only *trnH–psbA* harboured a unique sequence for each species. In the study of Newmaster et al. [83], the *matK* sequence was unique in half of the species investigated, and by combining the *matK* and *trnH-psbA* datasets, nearly 95% of the specimens could be identified successfully at the species level with a BLAST approach [83]. A number of other studies relying on *trnH–psbA* alone [56] or in combination with other regions [53], [58], [65] have confirmed the utility and efficacy of this region for plant barcoding [84]. However, in the genus *Fraxinus*, the *matK/trnH-psbA* combination was not better than using *trnH-psbA* alone, because *matK* sequences showed little polymorphism. In the shrub genus *Berberis*, Roy et al. [85] showed the uselessness of the *matK*, *rbcL* and *trhH-psbA* cpDNA regions for barcoding because of probable reticulate evolution, whereas in the genus *Quercus*, Piredda et al. [86] reported null discrimination power, because of low variation rate of the cpDNA regions investigated and additional biogeographical reasons. In the economically important timber genus *Cedrela*, no cpDNA barcode allowed a satisfactory identification of species; only the nITS showed correct identification for more than 50% species [60].

### Is there a universal and reliable cpDNA barcode for tree taxa?

Many other cpDNA loci have been developed and proposed for a standardised barcode (for a review, see [41]). However, as observed in our study, many did not yield good results for identifying tree species [41], [60]. Therefore, the simpler CBOL barcode [32], which is based on the conserved *rbcL* for anchoring plant groups and on a unique more variable locus, *matK*, for species identification, does not provide sufficient variation in many plant groups for the task of discriminating safely species, including *Fraxinus*. Considering our results and previously published studies focusing on tree or other woody genera, for instance in the Meliaceae where the CBOL protocol was largely inefficient [60], [87], we predict that simple DNA barcoding using one or a few loci will be inefficient for shrub or tree genera with similar population genetics attributes and speciation patterns as seen in *Fraxinus*, such as for *Picea*, in conifers [80]. As previously suggested [88], a nuclear barcode should be considered for these genera.

### Hopes and pitfalls of a nuclear barcode

The discovery of low-copy nuclear regions with sufficient genetic variability that are amplifiable with universal markers is difficult in plants because many, if not most of the nuclear genes are organized in multigene families [89], [90], [91] and because of the abundance of retrotransposons and other repetitive elements in the plant nuclear genome [92]. These features could result in amplification of paralogous sequences among taxa [93], [94] and poor PCR amplifications and sequencing quality in some groups [35]. A region that is commonly used with success in phylogenetic studies of land plants at the generic level is the nuclear ribosomal internal transcribed spacer region (nITS), which had been used early in studies on deciduous tree taxa (e.g. [50], [73]). Nuclear ITS sequences have been proposed as a barcode locus for plants for some time [31]. It was recently suggested as a additional marker by CBOL [32]. The use of ITS was validated as an efficient barcode locus for identifying species in many groups [30], [33], [34], [35], [60], [95], including ashes [35] and other tree genera such as *Cedrela* [60] and *Quercus* [86], whereas nITS did not always result in adequate discrimination of species in some genera of the Juglandaceae [96]. The presence of paralogous nITS sequences in some genera [97] may pose some problems for the universal use of nITS in plant barcoding. However in *Fraxinus*, nITS sequences have been used successfully to investigate the phylogeny of the genus [13], [73], as for many other angiosperm genera [50], [98], [99], [100], [101], [102]. Another potentially useful region for barcoding is the nuclear external transcribed spacer (nETS) [36]. It usually shows a high level of concerted evolution [39], with potentially useful polymorphisms deriving for the more or less rapid fixation of new variants within species [40].

In view of the present results, the adequate identification of *Fraxinus* species will result from the development and use of a multilocus barcode [32], [88], [103], [104], presumably including a more conserved cpDNA region for genus recognition, in conjunction with highly variable nuclear regions for species identification. Such a tiered approach has been advocated by CBOL [32] and Newmaster et al. [25], where a more conserved region (*rbcL*) is used first to establish the taxonomic group such as the generic or subgeneric assignment. Due to the lack of variation of *rbcL* to decipher sections or species in the genus *Fraxinus*, *trnH-psbA* appeared to be the most promising for this purpose, as outlined by Lahaye et al. [84] in a floristic inventory context. As for identifying *Fraxinus* species, the more variable region could be nITS, perhaps in combination with the nuclear external transcribed spacer (nETS), which is highly variable in the Oleaceae [105] and in *Fraxinus* [13].

### An endless search?

A simple and universal barcode for land plants probably represents a taxonomist's search for the Holy Grail [24], [106], in that probably no single cpDNA region will be variable enough, and nuclear loci will require primers specific to relatively small taxonomic groups, far from the efficiency and universality promoted by barcode initiators [12]. Moreover, even after controlling for the amount of parsimony-informative information available per species, the discrimination success will likely be lower in plants than in animals, given the high frequency of natural interspecific hybridization in plants [12].

The development of such a DNA barcode in the genus *Fraxinus* and for other tree taxa will require extensive amounts of additional sequence information at the genus level and in particular, for the nuclear genome. For example, the DNA barcoding efforts could take advantage of the completely sequenced genomes of *Arabidopsis*, *Populus*, *Oryza*, *Vitis*, and other species that are available in GenBank. Because in some cases, such as in the genus *Fraxinus* and likely in other tree taxa, regions of the genome thought to be neutral evolve too slowly to enable the recognition of cryptic or closely-related species pairs, large-scale genomics comparisons between closely-related species will be useful to identify regions under divergent selection, which could be involved in speciation [61], [107]. Moreover, a better knowledge of the comparative organisation of paralogous and orthologous genes in sequenced species pairs [108] will help construct gene catalogs and select promising regions that could match with the molecular barcode specifications. Given that comparative bioinformatic tools and databases become available to process efficiently such complex information at various levels of taxonomical diversity, technological progress will, in a “perhaps not so distant“ future, results in even more affordable prices for molecular determinations or for whole cpDNA genome sequences determined from single genomic molecules [109].

## Materials and Methods

### Species and loci sampling

We sampled 253 individuals from the wild, from arboreta, and from herbaria (between 2 and 28 individuals per species for 49 species, and 1 individual for each of seven other species), representative of the species diversity found in the genus *Fraxinus*. The sampling did not require any specific permits, as it was realized on government-owned sites.

We examined first the genetic variability in a preliminary subsample of 52 specimens representative of 23 species, hereafter called “reduced dataset”, using the two barcode options proposed by Chase et al. [42]. We then sequenced the complete dataset (253 individuals, hereafter called “expanded dataset”) for the most variable locus, and a complementary locus from Shaw et al. [62], identified as highly variable by preliminary tests (see below). For the expanded dataset, two highly variable chloroplast loci, the intergenic spacers *trnH-psbA* and *rpl32-trnL*, were sequenced and tested separately. The species analysed in this study are shown in Table S1. Taxa nomenclature and synonyms follow the taxonomical recommendations of Wallander [13] (Table 1).

### Molecular methods

For each sample, 25 µg of fresh leaves were dehydrated in an alcohol/acetone 70∶30 solution, and stored dry before extraction, following a modified protocol from Fernandez-Manjarres et al. [18]. This procedure allowed us to recover more DNA than using silica gel dried samples, due to the high level of phenols in *Fraxinus* leaves [110] (Raquin C., pers. comm.). DNA extraction was carried out using the DNeasy Plant Mini Kit (Qiagen) following manufacturer's instructions.

Four primer pairs targeting four regions of the chloroplast genome suggested by Chase et al. [42] were used: *matK*-F1/*matK*-R1, *rpoC1*-F1/*rpoC1*-R1, *rpoB*-F1/*rpoB*-R2 (available at http://www.kew.org/barcoding/protocols.html), and *trnH–psbA*F/*trnH–psbA*R [31]. *MatK*, *rpoC1*, *rpoB*, and *trnH-psbA* were sequenced for the reduced dataset, and *trnH-psbA* was sequenced for the expanded dataset. All protocols are available at http://www.kew.org/barcoding/protocols.html. In addition, in an effort to identify other potentially useful discriminating cpDNA regions for *Fraxinus*, we examined the level of sequence variation for the 21 cpDNA regions proposed by Shaw et al. [62] using a representative panel of 45 *Fraxinus* species. We performed preliminary tests for the five regions that showed the best normalized potentially informative character (PIC) (see Fig. 4 in [62]). Two of them resulted in clear amplification, and *rpl32-trnL* was the only one exhibiting variation among the samples analysed (results not shown). In the present study, this locus was further sequenced for all individuals of the expanded dataset, in addition to *trnH-psbA*. The primer sequences used for amplification, PCR conditions and DNA sequencing of this region were as described by Shaw et al. [62].

The annealing temperatures for *trnH–psbA* and *rpl32–trnL* were modified to 57°C and 56°C, respectively, to improve the efficiency of PCR. PCR was performed in a PTC-200 Thermal Cycler (MJ Research). The amplified PCR products were checked on 1.5% agarose gels. All DNA sequencing was performed at the Genoscope facilities at Centre National de Séquençage (91000 Evry, France). PCR products were purified using exonucleaseI and phosphatase, and sequenced using BigDyeTerminator V3.1 kit (Applied Biosystem) and a ABI3730XL sequencer. All regions were sequenced for both strands to confirm sequence accuracy. All new sequences have been deposited in GenBank under the accession numbers GU991679 to GU991721 (*rpoB*), HM130620 to HM130660 (*rpoC1*), HM171487 to HM171528 (*matK*), HM367360 to HM367586 (*trnH-psbA*) and HM222716 to HM222923 (*rpl32-trnL*).

### Numerical analyses

The quality of the sequences was checked using CodonCode Aligner version 1.6.3 (Codon Code Corporation, Dedham, MA, USA). Further alignments were performed using BioEdit [111] and with ClustalW [112] using default settings, followed by manual adjustments. Autapomorphic insertions or deletions in coding regions were treated as processing errors and deleted after rechecking of the chromatogram for both strands. The aligned portions of *rpoC1, rpoB, matK, and trnH–psbA* for all individuals of the reduced dataset were concatenated so as to test two different three-region barcodes proposed by Chase et al. [42], and hereafter designated as “option 1” (*rpoC1*, *rpoB* and *matK*) and “option 2” (*rpoC1*, *matK* and *trnH-psbA*). Because many studies [32], [57], [103] have shown variable PCR and sequencing success according to taxonomic groups and loci, it is likely that very few species in the Barcode of Life Data system (BOLD, [2]) will be represented for all the loci proposed as a standardised barcode. Nevertheless, it has been shown that adding sequences, even incomplete data for some taxa, can dramatically improve the delineation of groups of similar sequences, even in combined datasets [113], [114]. By considering the practical limitations to obtain three loci for all samples and the usefulness of incomplete data for some taxa, we chose to use all available data, independently of the number of loci successfully sequenced for each taxon.

Several methods have been used for the analysis of barcode data, including phylogenetic analysis [55], [56], [115], [116], [117], multidimensional graphics [53], [65], coalescent reconstruction of the genetic clusters [84], similarity approaches such as BLAST [23], [118] and approaches based on the ratio of minimum interspecific distance to maximum intraspecific distance [32], [119]. Irrespective of this variety of analytical approaches, it remains that the fundamental requirement for delimiting species is a level of interspecific polymorphism high enough to allow the grouping of individuals from the same species and the formation of distinct clusters at the interspecific level. Because it has been shown that the more robust and reliable method with different datasets was the “one nearest neighbour”, which relies on neighbor-joining (NJ) trees [120], we tested this approach as originally described in Hebert et al. [1] and suggested by Chase et al. [42], which implicates the estimation of the pairwise two-parameter substitution rates of Kimura [121] (K2P) proposed as a standard distance for barcoding animal taxa [1], in conjunction with the NJ algorithm of tree reconstruction [122]. The method has been reported as fast and accurate for both examining relationships among species and to assign unidentified samples to known species [1]. More complex methods of tree reconstruction exist (such as probabilistic trees obtained by maximum likelihood or Bayesian approaches) though they would not translate in better taxa discrimination if intraspecific divergence was equal or higher than interspecific divergence or if interspecific divergence was null [1], [123]. Using concatenated sequences and according to the protocol of Chase et al., [42], pairwise distances were estimated according to the K2P model and NJ trees (implemented in the BOLD website as a “taxon ID tree” integrated analytics, see [2]) were estimated using PAUP version 4.0 [124]. Bootstrap analyses were based on 1000 replicates in all cases. *Jasminum nudiflorum* was used as the outgroup (sequence from [125]). The same analyses were conducted independently for the expanded dataset (*trnH–psbA* and *rpl32–trnL*). We considered that a locus, or a concatenation of loci, accurately discriminated a species when more than 50% of the individuals sampled fell in the same monophyletic group. This relatively low threshold has been chosen to reflect the minimum probability for which a correct identification would be more likely than a wrong identification. In some cases, samples were classified as misidentified with a high level of confidence. Those cases occurred when a sample from a given taxon showed so many substitutions that it would be classified further away than being a sister group to its conspecifics, sometimes in a different section, even after carefully rechecking these individuals. We chose to note them as “misidentified”, to reflect the fact that, despite all the careful checks in the barcoding process, a misidentification could occur.

BLAST was tested as an alternative to the previous approach. BLAST is already used in large databases, such as GenBank, and reportedly discriminates more accurately sequences with low divergence [2], [23], [118]. As a test case, we built a BLAST database with default parameters in BioEdit using the *trnH–psbA* sequences obtained for the expanded dataset, which corresponded to the most variable cpDNA locus proposed by CBOL [42]. A database BLAST search was then conducted for each individual sequence and the first hit for a successful identification was checked. To avoid artifactual auto-BLAST results (when a BLAST result corresponds to the sequence itself), the sequence used for the BLAST query was removed manually from the results, and unidentified samples were not included.

To assess the discriminatory power of the different barcode options as measured by the size of the gap between the distributions of intraspecific and interspecific genetic distances, interspecific and intraspecific K2P genetic distances were calculated for the options 1 and 2, *matK*, *trnH-psbA,* and *rpl32-trnL* using PAUP version 4.0 [124]. The taxa represented by only one sample were not considered for the calculation of intraspecific distances.

## Supporting Information

Table S1 *Fraxinus* samples used in this study, herbarium vouchers, and newly published DNA sequences. ID stands for identifier. Sample type related to the origin of the samples: A, arboretum; W, wild collected; H, herbarium. Vouchers are deposited at the National Herbarium, Muséum National d'Histoire Naturelle, Paris, France (P00729547 to P00729694), or at the Mexico Herbarium (MEXU1032796 to MEXU991880).
(DOC)
