# Supplementary material for: Deciduous Trees and the Application of Universal DNA Barcodes: A Case Study on the Circumpolar Fraxinus
Source: PLoS One. 2012 Mar 27;7(3):e34089. doi: 10.1371/journal.pone.0034089 (PMC3313964; doi:10.1371/journal.pone.0034089)
Supplement: Table S1 — Fraxinus samples used in this study, herbarium vouchers, and newly published DNA sequences. ID stands for identifier. Sample type related to the origin of the samples: A, arboretum; W, wild collected; H, herbarium. Vouchers are deposited at the National Herbarium, Muséum National d'Histoire Naturelle, Paris, France (P00729547 to P00729694), or at the Mexico Herbarium (MEXU1032796 to MEXU991880). (DOC) [file pone.0034089.s001.doc]

| Taxa | Sample type | Vouchers ID | Botanical garden ID | Sample ID | Origin | GenBank accession numbers | | | | |
| --- | --- | --- | --- | --- | --- | --- | --- | --- | --- | --- |
|  |  |  |  |  |  | *rpoB* | *rpoC* | *matK* | *trnH-psbA* | *rpl32-trnL* |
| *Fraxinus americana* | A | P00729688 | 15 | 2 | Arboretum National des Barres (Nogent-sur-Vernisson, France) | HM130620 | GU991679 | HM171496 | HM367363 | HM222718 |
| *Fraxinus americana* | A |  | 1137 | 35 | Arboretum National des Barres (Nogent-sur-Vernisson, France) | HM130621 | |  | HM367364 | HM222719 |
| *Fraxinus americana* | A | P00729667 |  | 117 | Pépinière Adeline (La Chapelle Montlinard, France) |  |  |  | HM367362 | HM222717 |
| *Fraxinus americana* 'Automn purple' | A | P00729638 | 2793 Kipling Street | 261 | Canopy Association (Palo Alto, U.S.A.) |  |  |  | HM367360 | HM222716 |
| *Fraxinus americana* 'Automn purple' | A | P00729636 | 598 Loma Verde Avenue | 262 | Canopy Association (Palo Alto, U.S.A.) |  |  |  | HM367361 |  |
| *Fraxinus americana* var. *biltmoreana* | A |  | 3826 | 45 | Arboretum National des Barres (Nogent-sur-Vernisson, France) | HM130625 | GU991682 | HM171519 | | HM222720 |
| *Fraxinus angustifolia* | A | P00729660 | 1877 | 17 | Arboretum National des Barres (Nogent-sur-Vernisson, France) |  |  | HM171493 | HM367373 | HM222730 |
| *Fraxinus angustifolia* | A |  | 1929 | 20 | Arboretum National des Barres (Nogent-sur-Vernisson, France) | HM130652 | GU991709 | HM171497 | HM367374 | HM222731 |
| *Fraxinus angustifolia* | A | P00729574 | 1987.1215*A | 214 | Sir Harold Hillier Garden (Romsey, U.K.) |  |  |  | HM367366 | HM222723 |
| *Fraxinus angustifolia* | A | P00729572 | 1991.0384*A | 215 | Sir Harold Hillier Garden (Romsey, U.K.) |  |  |  | HM367365 | HM222721 |
| *Fraxinus angustifolia* | W |  |  | Portugal1 |  | HM130622 | GU991680 | HM171527 | |  |
| *Fraxinus angustifolia* ssp. *oxycarpa* | A |  | 1731-1991 | 251 | Jardin botanique de Montréal (Montréal, Canada) |  |  |  | HM367367 | HM222724 |
| *Fraxinus angustifolia* var. *monophylla* | A |  | 1979.0263*A | 233 | Sir Harold Hillier Garden (Romsey, U.K.) |  |  |  |  | HM222722 |
| *Fraxinus anomala* | A |  | B0702 | 284 | Parc Botanique du Launay (Orsay, France) |  |  |  | HM367379 | HM222738 |
| *Fraxinus anomala* | A |  | B0703 | 285 | Parc Botanique du Launay (Orsay, France) |  |  |  | HM367380 | HM222739 |
| *Fraxinus apertisquamifera* | W | P00729616 | 1 | aperti1 | leg Kazuya Iizuka (Utsunomiya University, Japan) |  |  |  | HM367381 | HM222740 |
| *Fraxinus apertisquamifera* | W | P00729614 | 2 | aperti2 | leg Kazuya Iizuka (Utsunomiya University, Japan) |  |  |  | HM367382 | HM222741 |
| *Fraxinus apertisquamifera* | W | P00729612 | 3 | aperti3 | leg Kazuya Iizuka (Utsunomiya University, Japan) |  |  |  | HM367383 | HM222742 |
| *Fraxinus apertisquamifera* | W | P00729610 | 4 | aperti4 | leg Kazuya Iizuka (Utsunomiya University, Japan) |  |  |  | HM367384 | HM222743 |
| *Fraxinus apertisquamifera* | W | P00729608 | 5 | aperti5 | leg Kazuya Iizuka (Utsunomiya University, Japan) |  |  |  | HM367385 | HM222744 |
| *Fraxinus apertisquamifera* | W | P00729606 | 6 | aperti6 | leg Kazuya Iizuka (Utsunomiya University, Japan) |  |  |  | HM367386 | HM222745 |
| *Fraxinus apertisquamifera* | W | P00729602 | 8 | aperti8 | leg Kazuya Iizuka (Utsunomiya University, Japan) |  |  |  | HM367387 | HM222746 |
| *Fraxinus apertisquamifera* | W | P00729600 | 9 | aperti9 | leg Kazuya Iizuka (Utsunomiya University, Japan) |  |  |  | HM367388 | HM222747 |
| *Fraxinus berlanderiana* | A | P00729670 | 1638 | 14 | Arboretum National des Barres (Nogent-sur-Vernisson, France) | HM130623 | GU991681 | |  | HM222748 |
| *Fraxinus berlanderiana* | A | P00729666 | 3617 | 48 | Arboretum National des Barres (Nogent-sur-Vernisson, France) | HM130624 | GU991722 | HM171521 | | HM222751 |
| *Fraxinus berlanderiana* | H | MEXU991880 | 991880 | 19 | Mexico Herbarium (Mexico, Mexico) |  |  |  | HM367390 | HM222749 |
| *Fraxinus berlanderiana* | H | MEXU1158611 | 1158611 | 17 | Mexico Herbarium (Mexico, Mexico) |  |  |  | HM367389 |  |
| *Fraxinus berlanderiana* | A | P00729567 | 1977.1152*Y | 225 | Sir Harold Hillier Garden (Romsey, U.K.) |  |  |  | HM367391 | HM222750 |
| *Fraxinus biltmoreana* | A | P00729569 | 1977.2370*Q | 226 | Sir Harold Hillier Garden (Romsey, U.K.) |  |  |  |  | HM222753 |
| *Fraxinus biltmoreana* | A |  |  | 121 | Arboretum Bayreuth (Bayreuth, Deutschland) |  |  |  | HM367393 |  |
| *Fraxinus biltmoreana* | A | P00729626 |  | 101 | Pépinière Adeline (La Chapelle Montlinard, France) |  |  |  | HM367392 | HM222752 |
| *Fraxinus bungeana* | A | P00729682 | 3811 | 41 | Arboretum National des Barres (Nogent-sur-Vernisson, France) |  | GU991683 | HM171516 | HM367395 | HM222755 |
| *Fraxinus bungeana* | A | P00729671 |  | 69 | Arboretum Chèvreloup (Rocquencourt, France) |  |  |  | HM367396 | HM222756 |
| *Fraxinus bungeana* | A | P00729618 |  | 106 | Pépinière Adeline (La Chapelle Montlinard, France) |  |  |  | HM367394 | HM222754 |
| *Fraxinus caroliniana* | A |  | 1980.0206*A | 202 | Sir Harold Hillier Garden (Romsey, U.K.) |  |  |  | HM367397 | HM222757 |
| *Fraxinus caroliniana* | A | P00729639 |  | 53 | Arboretum Chèvreloup (Rocquencourt, France) |  |  |  | HM367398 | HM222758 |
| *Fraxinus chiisanensis* | A |  | 8907 | 24 | Arboretum National des Barres (Nogent-sur-Vernisson, France) | HM130627 | GU991685 | HM171501 | HM367401 | HM222761 |
| *Fraxinus chiisanensis* | A | P00729662 | 8994 | 21 | Arboretum National des Barres (Nogent-sur-Vernisson, France) | HM130626 | GU991684 | HM171498 | HM367399 | HM222759 |
| *Fraxinus chiisanensis* | A | P00729575 | 2006.0536*A | 230 | Sir Harold Hillier Garden (Romsey, U.K.) |  |  |  | HM367400 | HM222760 |
| *Fraxinus chinensis* | A | P00729641 | 1992 | 31 | Arboretum National des Barres (Nogent-sur-Vernisson, France) | HM130629 | GU991686 | HM171509 | HM367405 | HM222765 |
| *Fraxinus chinensis* | A | P00729578 | 1966.2754*A | 219 | Sir Harold Hillier Garden (Romsey, U.K.) |  |  |  | HM367404 | HM222764 |
| *Fraxinus chinensis* | A |  | 1982.2663*A | 210 | Sir Harold Hillier Garden (Romsey, U.K.) |  |  |  | HM367403 | HM222763 |
| *Fraxinus chinensis* | A | P00729649 |  | 108 | Pépinière Adeline (La Chapelle Montlinard, France) |  |  |  | HM367402 | HM222762 |
| *Fraxinus chinensis* ssp. *rhyncophylla* | A | P00729588 | 2140 | 26 | Arboretum National des Barres (Nogent-sur-Vernisson, France) | HM130628 | | HM171503 | HM367408 | HM222768 |
| *Fraxinus chinensis* ssp. *rhyncophylla* | A | P00729656 | 3815 | 42 | Arboretum National des Barres (Nogent-sur-Vernisson, France) | HM130630 | GU991687 | | HM367409 | HM222769 |
| *Fraxinus chinensis* ssp. *rhyncophylla* | A | P00729577 | 2001.0366*A | 229 | Sir Harold Hillier Garden (Romsey, U.K.) |  |  |  | HM367407 | HM222767 |
| *Fraxinus chinensis* ssp. *rhyncophylla* | A | P00729651 |  | 109 | Pépinière Adeline (La Chapelle Montlinard, France) |  |  |  | HM367406 | HM222766 |
| *Fraxinus cuspidata* | H | MEXU658827 | 658827 | 9 | Mexico Herbarium (Mexico, Mexico) |  |  |  | HM367414 | HM222774 |
| *Fraxinus cuspidata* | H | MEXU758471 | 758471 | 2 | Mexico Herbarium (Mexico, Mexico) |  |  |  | HM367410 | HM222770 |
| *Fraxinus cuspidata* | A |  |  | 290 | Parc Botanique du Launay (Orsay, France) |  |  |  | HM367413 | HM222773 |
| *Fraxinus cuspidata* | A | P00729601 |  | 288 | Peckerwood Garden (Hempstead, U.S.A.) |  |  |  | HM367411 | HM222771 |
| *Fraxinus cuspidata* | A | P00729603 |  | 289 | Peckerwood Garden (Hempstead, U.S.A.) |  |  |  | HM367412 | HM222772 |
| *Fraxinus dipetala* | A | P00729560 | 10303*A6 | 245 | Rancho Santa Anna Bot. Garden (Claremont, U.S.A.) |  |  |  | HM367415 | HM222776 |
| *Fraxinus dipetala* | A | P00729548 | 13476*D2 | 243 | Rancho Santa Anna Bot. Garden (Claremont, U.S.A.) |  |  |  |  | HM222775 |
| *Fraxinus dipetala* | A | P00729633 |  | 56 | Arboretum Chèvreloup (Rocquencourt, France) |  |  |  |  | HM222777 |
| *Fraxinus dipetala* | A | P00729621 |  | 65 | Arboretum Chèvreloup (Rocquencourt, France) |  |  |  | HM367416 | HM222778 |
| *Fraxinus excelsior* | A | P00729583 | 1977.0646 | 232 | Sir Harold Hillier Garden (Romsey, U.K.) |  |  |  | HM367418 |  |
| *Fraxinus excelsior* | A |  |  | TF | Parc Botanique du Launay (Orsay, France) | HM130632 | GU991688 | HM171528 | HM367419 |  |
| *Fraxinus excelsior* | A | P00729561 |  | 228 | Sir Harold Hillier Garden (Romsey, U.K.) |  |  |  | HM367417 | HM222779 |
| *Fraxinus excelsior monophylla* | A | P00729664 | 3263 | 50 | Arboretum National des Barres (Nogent-sur-Vernisson, France) | HM130633 | | HM171524 | HM367421 | HM222781 |
| *Fraxinus excelsior monophylla* | A | P00729576 |  | 221 | Sir Harold Hillier Garden (Romsey, U.K.) |  |  |  | HM367420 | HM222780 |
| *Fraxinus excelsior* var. *nana* | A |  | 3337 | 49 | Arboretum National des Barres (Nogent-sur-Vernisson, France) | HM130634 | | HM171522 | HM367422 | HM222782 |
| *Fraxinus excelsior* var. *pendula* | A | P00729652 | 350 | 11 | Arboretum National des Barres (Nogent-sur-Vernisson, France) | HM130631 | GU991689 | HM171489 | HM367423 | HM222783 |
| *Fraxinus floribunda* | A | P00729680 | 1739 | 16 | Arboretum National des Barres (Nogent-sur-Vernisson, France) | HM130651 | GU991708 | HM171492 | | HM222869 |
| *Fraxinus floribunda* | A | P00729571 | 1977.0702*Y | 223 | Sir Harold Hillier Garden (Romsey, U.K.) |  |  |  | HM367425 | HM222785 |
| *Fraxinus floribunda* | A | P00729586 | 1977.0718*S | 222 | Sir Harold Hillier Garden (Romsey, U.K.) |  |  |  | HM367424 | HM222784 |
| *Fraxinus floribunda* | A | P00729645 | 2162 | 27 | Arboretum National des Barres (Nogent-sur-Vernisson, France) |  | GU991707 | HM171504 | HM367535 | HM222870 |
| *Fraxinus formosana* | A | P00729591 | OSN84-00-0285-60 | 248 | Botanic Garden Osnabrück (Osnabrück, Deutschland) |  |  |  | HM367426 |  |
| *Fraxinus gooddingii* | H | MEXU1214941 | 1214941 | 5 | Mexico Herbarium (Mexico, Mexico) |  |  |  | HM367427 | HM222786 |
| *Fraxinus greggii* | A | P00729693 | 801 | 281 | University of Arizona Campus Arboretum (Tucson, U.S.A.) |  |  |  | HM367430 | HM222788 |
| *Fraxinus greggii* | H | MEXU529898 | 529898 | 14 | Mexico Herbarium (Mexico, Mexico) |  |  |  | HM367428 |  |
| *Fraxinus greggii* | A |  | 19860253 | 246 | Cambridge University Botanic Garden (Cambridge, U.K) |  |  |  | HM367429 | HM222787 |
| *Fraxinus greggii* | A | P00729605 |  | 294 | Peckerwood Garden (Hempstead, U.S.A.) |  |  |  | HM367431 | HM222789 |
| *Fraxinus greggii* | A | P00729607 |  | 296 | Peckerwood Garden (Hempstead, U.S.A.) |  |  |  | HM367433 |  |
| *Fraxinus greggii* | A | P00729609 |  | 295 | Peckerwood Garden (Hempstead, U.S.A.) |  |  |  | HM367432 | HM222790 |
| *Fraxinus griffithii* | A | P00729581 | 1977.2517*T | 234 | Sir Harold Hillier Garden (Romsey, U.K.) |  |  |  | HM367434 | HM222791 |
| *Fraxinus griffithii* | A |  | 783-01 | 276 | Parc Botanique du Launay (Orsay, France) |  |  |  | HM367435 | HM222792 |
| *Fraxinus griffithii* | A |  | 783-10 | 277 | Parc Botanique du Launay (Orsay, France) |  |  |  | HM367436 |  |
| *Fraxinus guilinensis* | A | P00729675 |  | 68 | Arboretum Chèvreloup (Rocquencourt, France) |  |  |  | HM367437 | HM222793 |
| *Fraxinus holotricha* | A | P00729668 | 3819 | 43 | Arboretum National des Barres (Nogent-sur-Vernisson, France) | HM130635 | GU991690 | HM171517 | HM367440 | HM222795 |
| *Fraxinus holotricha* | A |  | 3820 | 44 | Arboretum National des Barres (Nogent-sur-Vernisson, France) |  | GU991691 | HM171518 | HM367441 | HM222796 |
| *Fraxinus holotricha* | A |  | 1982.0066*A | 209 | Sir Harold Hillier Garden (Romsey, U.K.) |  |  |  | HM367439 |  |
| *Fraxinus holotricha* | A | P00729628 |  | 102 | Pépinière Adeline (La Chapelle Montlinard, France) |  |  |  | HM367438 | HM222794 |
| *Fraxinus hoopiensis* | A |  | 19990031 | 283 | Arboretum Kalmthout (Kalmthout,Netherland) |  |  |  |  | HM222797 |
| *Fraxinus incana* | A |  |  | 122 | Arboretum Bayreuth (Bayreuth, Deutschland) |  |  |  | HM367442 |  |
| *Fraxinus lanuginosa* | W |  | 107 | 137 | Forestry and Forest Products Research Institute (Tsukuba, Japan) |  |  |  | HM367443 | HM222798 |
| *Fraxinus lanuginosa* | W |  | 107 | 164 | Forestry and Forest Products Research Institute (Tsukuba, Japan) |  |  |  | HM367445 | HM222799 |
| *Fraxinus lanuginosa* | W |  | 108 | 138 | Forestry and Forest Products Research Institute (Tsukuba, Japan) |  |  |  | HM367444 |  |
| *Fraxinus lanuginosa* | W |  | 108 | 165 | Forestry and Forest Products Research Institute (Tsukuba, Japan) |  |  |  | HM367446 |  |
| *Fraxinus lanuginosa* | W |  | 274 | 188 | Forestry and Forest Products Research Institute (Tsukuba, Japan) |  |  |  | HM367447 |  |
| *Fraxinus lanuginosa* | W |  | 275 | 189 | Forestry and Forest Products Research Institute (Tsukuba, Japan) |  |  |  | HM367448 |  |
| *Fraxinus lanuginosa* | W |  | 276 | 190 | Forestry and Forest Products Research Institute (Tsukuba, Japan) |  |  |  | HM367449 |  |
| *Fraxinus lanuginosa* var. *serrata* | W |  | 78 | 134 | Forestry and Forest Products Research Institute (Tsukuba, Japan) |  |  |  | HM367450 | HM222800 |
| *Fraxinus lanuginosa* var. *serrata* | W |  | 83 | 135 | Forestry and Forest Products Research Institute (Tsukuba, Japan) |  |  |  | HM367451 | HM222801 |
| *Fraxinus lanuginosa* var. *serrata* | W |  | 97 | 136 | Forestry and Forest Products Research Institute (Tsukuba, Japan) |  |  |  | HM367452 | HM222802 |
| *Fraxinus lanuginosa* var. *serrata* | W |  | 113 | 139 | Forestry and Forest Products Research Institute (Tsukuba, Japan) |  |  |  | HM367453 | HM222803 |
| *Fraxinus lanuginosa* var. *serrata* | W |  | 132 | 140 | Forestry and Forest Products Research Institute (Tsukuba, Japan) |  |  |  | HM367454 | HM222804 |
| *Fraxinus lanuginosa* var. *serrata* | W |  | 139 | 141 | Forestry and Forest Products Research Institute (Tsukuba, Japan) |  |  |  | HM367455 | HM222805 |
| *Fraxinus lanuginosa* var. *serrata* | W |  | 165 | 142 | Forestry and Forest Products Research Institute (Tsukuba, Japan) |  |  |  | HM367456 | HM222806 |
| *Fraxinus lanuginosa* var. *serrata* | W |  | 170 | 143 | Forestry and Forest Products Research Institute (Tsukuba, Japan) |  |  |  | HM367457 | HM222807 |
| *Fraxinus lanuginosa* var. *serrata* | W |  | 176 | 144 | Forestry and Forest Products Research Institute (Tsukuba, Japan) |  |  |  | HM367458 | HM222808 |
| *Fraxinus lanuginosa* var. *serrata* | W |  | 181 | 145 | Forestry and Forest Products Research Institute (Tsukuba, Japan) |  |  |  | HM367459 | HM222809 |
| *Fraxinus lanuginosa* var. *serrata* | W |  | 185 | 146 | Forestry and Forest Products Research Institute (Tsukuba, Japan) |  |  |  | HM367460 | HM222810 |
| *Fraxinus lanuginosa* var. *serrata* | W |  | 188 | 147 | Forestry and Forest Products Research Institute (Tsukuba, Japan) |  |  |  | HM367461 | HM222811 |
| *Fraxinus lanuginosa* var. *serrata* | W |  | 191 | 148 | Forestry and Forest Products Research Institute (Tsukuba, Japan) |  |  |  | HM367462 | HM222812 |
| *Fraxinus lanuginosa* var. *serrata* | W |  | 192 | 149 | Forestry and Forest Products Research Institute (Tsukuba, Japan) |  |  |  | HM367463 | HM222813 |
| *Fraxinus lanuginosa* var. *serrata* | W |  | 194 | 150 | Forestry and Forest Products Research Institute (Tsukuba, Japan) |  |  |  | HM367464 | HM222814 |
| *Fraxinus lanuginosa* var. *serrata* | W |  | 218 | 152 | Forestry and Forest Products Research Institute (Tsukuba, Japan) |  |  |  | HM367465 | HM222815 |
| *Fraxinus lanuginosa* var. *serrata* | W |  | 238 | 153 | Forestry and Forest Products Research Institute (Tsukuba, Japan) |  |  |  | HM367466 | HM222816 |
| *Fraxinus latifolia* | A |  | 408 | 3 | Arboretum National des Barres (Nogent-sur-Vernisson, France) | HM130636 | | HM171507 | HM367469 | HM222820 |
| *Fraxinus latifolia* | A |  | 3808 | 40 | Arboretum National des Barres (Nogent-sur-Vernisson, France) | HM130637 | GU991692 | HM171515 | HM367470 | HM222821 |
| *Fraxinus latifolia* | A | P00729615 | 10501*C1 | 237 | Rancho Santa Anna Bot. Garden (Claremont, U.S.A.) |  |  |  | HM367467 | HM222817 |
| *Fraxinus latifolia* | A | P00729554 | 10521*A3 | 244 | Rancho Santa Anna Bot. Garden (Claremont, U.S.A.) |  |  |  | HM367468 | HM222819 |
| *Fraxinus latifolia* | A | P00729552 | 8669*A4 | 238 | Rancho Santa Anna Bot. Garden (Claremont, U.S.A.) |  |  |  |  | HM222818 |
| *Fraxinus latifolia* | A | P00729619 |  | 66 | Arboretum Chèvreloup (Rocquencourt, France) |  |  |  | HM367471 | HM222822 |
| *Fraxinus longicuspis* | W |  | 1 | 154 | Forestry and Forest Products Research Institute (Tsukuba, Japan) |  |  |  | HM367474 | HM222824 |
| *Fraxinus longicuspis* | W |  | 3 | 156 | Forestry and Forest Products Research Institute (Tsukuba, Japan) |  |  |  | HM367475 | HM222825 |
| *Fraxinus longicuspis* | W |  | 4 | 157 | Forestry and Forest Products Research Institute (Tsukuba, Japan) |  |  |  | HM367476 |  |
| *Fraxinus longicuspis* | W |  | 5 | 158 | Forestry and Forest Products Research Institute (Tsukuba, Japan) |  |  |  | HM367477 | HM222826 |
| *Fraxinus longicuspis* | W |  | 6 | 159 | Forestry and Forest Products Research Institute (Tsukuba, Japan) |  |  |  | HM367478 | HM222827 |
| *Fraxinus longicuspis* | W |  | 7 | 160 | Forestry and Forest Products Research Institute (Tsukuba, Japan) |  |  |  | HM367479 | HM222828 |
| *Fraxinus longicuspis* | W |  | 8 | 161 | Forestry and Forest Products Research Institute (Tsukuba, Japan) |  |  |  | HM367480 |  |
| *Fraxinus longicuspis* | W |  | 9 | 162 | Forestry and Forest Products Research Institute (Tsukuba, Japan) |  |  |  | HM367481 | HM222829 |
| *Fraxinus longicuspis* | W |  | 10 | 163 | Forestry and Forest Products Research Institute (Tsukuba, Japan) |  |  |  | HM367482 |  |
| *Fraxinus longicuspis* | A | P00729690 | 2083 | 25 | Arboretum National des Barres (Nogent-sur-Vernisson, France) | HM130638 | GU991693 | HM171502 | HM367483 | HM222830 |
| *Fraxinus longicuspis* | A |  |  | 123 | Arboretum Bayreuth (Bayreuth, Deutschland) |  |  |  | HM367473 |  |
| *Fraxinus longicuspis* | A | P00729653 |  | 110 | Pépinière Adeline (La Chapelle Montlinard, France) |  |  |  | HM367472 | HM222823 |
| *Fraxinus longicuspis* var. *latifolia* | A |  | 1438-1972 | 256 | Jardin botanique de Montréal (Montréal, Canada) |  |  |  | HM367484 | HM222831 |
| *Fraxinus malacophylla* | A | P00729640 |  | 273 | Colvos Creek Nursery (Seattle, U.S.A.) |  |  |  | HM367486 |  |
| *Fraxinus malcophylla* | A | P00729564 | 1969-1384-P | 264 | Los Angeles County Arboretum and Botanic Garden (Los Angeles, U.S.A.) | |  |  | HM367485 |  |
| *Fraxinus mandshurica* | A | P00729677 | 2456 | 22 | Arboretum National des Barres (Nogent-sur-Vernisson, France) |  | GU991694 | HM171499 | HM367489 | HM222834 |
| *Fraxinus mandshurica* | A | P00729590 | 2500 | 23 | Arboretum National des Barres (Nogent-sur-Vernisson, France) | HM130639 | GU991695 | HM171500 | HM367490 | HM222835 |
| *Fraxinus mandshurica* | A | P00729559 | 1982.2662*A | 212 | Sir Harold Hillier Garden (Romsey, U.K.) |  |  |  | HM367488 | HM222833 |
| *Fraxinus mandshurica* | A | P00729620 |  | 105 | Pépinière Adeline (La Chapelle Montlinard, France) |  |  |  | HM367487 | HM222832 |
| *Fraxinus mandshurica* var. *japonica* | W |  | 263 | 184 | Forestry and Forest Products Research Institute (Tsukuba, Japan) |  |  |  | HM367491 |  |
| *Fraxinus mandshurica* var. *japonica* | W |  | 264 | 185 | Forestry and Forest Products Research Institute (Tsukuba, Japan) |  |  |  | HM367492 |  |
| *Fraxinus mandshurica* var. *japonica* | W |  | 265 | 186 | Forestry and Forest Products Research Institute (Tsukuba, Japan) |  |  |  | HM367493 |  |
| *Fraxinus mandshurica* var. *japonica* | W |  | 296 | 192 | Forestry and Forest Products Research Institute (Tsukuba, Japan) |  |  |  | HM367494 |  |
| *Fraxinus mandshurica* var. *japonica* | W |  | 297 | 193 | Forestry and Forest Products Research Institute (Tsukuba, Japan) |  |  |  | HM367495 |  |
| *Fraxinus mariesii* | A | P00729679 | 1883 | 18 | Arboretum National des Barres (Nogent-sur-Vernisson, France) | HM130640 | GU991696 | HM171494 | | HM222836 |
| *Fraxinus micrantha* | A |  |  | 292 | Parc Botanique du Launay (Orsay, France) |  |  |  |  | HM222837 |
| *Fraxinus micrantha* | A |  |  | 293 | Parc Botanique du Launay (Orsay, France) |  |  |  | HM367496 | HM222838 |
| *Fraxinus nigra* | A | P00729686 | 1994 | 33 | Arboretum National des Barres (Nogent-sur-Vernisson, France) | HM130641 | GU991697 | | HM367498 | HM222840 |
| *Fraxinus nigra* | A | P00729632 | 3828 | 47 | Arboretum National des Barres (Nogent-sur-Vernisson, France) | HM130642 | GU991698 | |  |  |
| *Fraxinus nigra* | A |  |  | 100 | Pépinière Adeline (La Chapelle Montlinard, France) |  |  |  | HM367497 | HM222839 |
| *Fraxinus obliqua* | A | P00729547 | 1982.3212*A | 207 | Sir Harold Hillier Garden (Romsey, U.K.) |  |  |  | HM367499 | HM222841 |
| *Fraxinus ornus* | A |  | 741 | 1 | Arboretum National des Barres (Nogent-sur-Vernisson, France) | HM130643 | GU991699 | HM171487 | | HM222842 |
| *Fraxinus ornus* | A |  | 1597 | 12 | Arboretum National des Barres (Nogent-sur-Vernisson, France) | HM130644 | GU991700 | HM171490 | HM367500 | HM222843 |
| *Fraxinus ornus* | A |  | 1887 | 19 | Arboretum National des Barres (Nogent-sur-Vernisson, France) | HM130645 | GU991701 | HM171495 | HM367501 | HM222844 |
| *Fraxinus ornus* | W |  | MOSOR1 | 286 | Leg Yakovlev |  |  |  | HM367503 |  |
| *Fraxinus ornus* | W |  | MOSOR2 | 287 | Leg Yakovlev |  |  |  | HM367504 |  |
| *Fraxinus ornus* | A | P00729672 |  | 58 | Arboretum Chèvreloup (Rocquencourt, France) |  |  |  | HM367505 | HM222845 |
| *Fraxinus ornus* | A | P00729644 |  | 274 | Colvos Creek Nursery (Seattle, U.S.A.) |  |  |  | HM367502 |  |
| *Fraxinus pallisiae* | A |  | 1034-1976 | 252 | Jardin botanique de Montréal (Montréal, Canada) |  |  |  | HM367371 | HM222728 |
| *Fraxinus pallisiae* | A | P00729570 | 1982.0951*A | 217 | Sir Harold Hillier Garden (Romsey, U.K.) |  |  |  | HM367369 | HM222726 |
| *Fraxinus pallisiae* | A | P00729584 | 1982.0951*B | 218 | Sir Harold Hillier Garden (Romsey, U.K.) |  |  |  | HM367370 | HM222727 |
| *Fraxinus pallisiae* | A | P00729624 |  | 103 | Pépinière Adeline (La Chapelle Montlinard, France) |  |  |  | HM367368 | HM222725 |
| *Fraxinus papillosa* | A | P00729692 | 1541 | 280 | University of Arizona Campus Arboretum (Tucson, U.S.A.) |  |  |  | HM367507 | HM222847 |
| *Fraxinus papillosa* | H | MEXU714886 | 714886 | 22 | Mexico Herbarium (Mexico, Mexico) |  |  |  |  | HM222846 |
| *Fraxinus paxiana* | A | P00729629 |  | 59 | Arboretum Chèvreloup (Rocquencourt, France) |  |  |  | HM367509 | HM222849 |
| *Fraxinus paxiana* | A | P00729556 |  | 60 | Arboretum Chèvreloup (Rocquencourt, France) |  |  |  | HM367510 | HM222850 |
| *Fraxinus paxiana* | A | P00729657 |  | 112 | Pépinière Adeline (La Chapelle Montlinard, France) |  |  |  | HM367508 | HM222848 |
| *Fraxinus pennsylvanica* | A | P00729622 |  | 104 | Pépinière Adeline (La Chapelle Montlinard, France) |  |  |  | HM367511 | HM222851 |
| *Fraxinus pennsylvanica* ssp. *pennsylvanica* | A | P00729648 | 1609 | 13 | Arboretum National des Barres (Nogent-sur-Vernisson, France) | HM130646 | GU991702 | HM171491 | | HM222852 |
| *Fraxinus pennsylvanica* ssp. *pennsylvanica* | A | P00729678 | 1639 | 15 | Arboretum National des Barres (Nogent-sur-Vernisson, France) |  | GU991703 | |  | HM222853 |
| *Fraxinus pennsylvanica* ssp. *pennsylvanica* | A |  | 3806 | 38 | Arboretum National des Barres (Nogent-sur-Vernisson, France) | HM130647 | GU991704 | | HM367512 | HM222854 |
| *Fraxinus platypoda* | W |  | 253 | 174 | Forestry and Forest Products Research Institute (Tsukuba, Japan) |  |  |  | HM367514 | HM222856 |
| *Fraxinus platypoda* | W |  | 254 | 175 | Forestry and Forest Products Research Institute (Tsukuba, Japan) |  |  |  | HM367515 | HM222857 |
| *Fraxinus platypoda* | W |  | 255 | 176 | Forestry and Forest Products Research Institute (Tsukuba, Japan) |  |  |  | HM367516 | HM222858 |
| *Fraxinus platypoda* | W |  | 256 | 177 | Forestry and Forest Products Research Institute (Tsukuba, Japan) |  |  |  | HM367517 |  |
| *Fraxinus platypoda* | W |  | 257 | 178 | Forestry and Forest Products Research Institute (Tsukuba, Japan) |  |  |  | HM367518 |  |
| *Fraxinus platypoda* | W |  | 258 | 179 | Forestry and Forest Products Research Institute (Tsukuba, Japan) |  |  |  | HM367519 |  |
| *Fraxinus platypoda* | W |  | 259 | 180 | Forestry and Forest Products Research Institute (Tsukuba, Japan) |  |  |  | HM367520 |  |
| *Fraxinus platypoda* | W |  | 260 | 181 | Forestry and Forest Products Research Institute (Tsukuba, Japan) |  |  |  | HM367521 |  |
| *Fraxinus platypoda* | W |  | 261 | 182 | Forestry and Forest Products Research Institute (Tsukuba, Japan) |  |  |  | HM367522 |  |
| *Fraxinus platypoda* | W |  | 262 | 183 | Forestry and Forest Products Research Institute (Tsukuba, Japan) |  |  |  | HM367523 |  |
| *Fraxinus platypoda* | A |  | 19810042 | 250 | Jard. Bot. Nat. de Belgique (Meise, Belgium) |  |  |  |  | HM222859 |
| *Fraxinus platypoda* | A | P00729635 |  | 55 | Arboretum Chèvreloup (Rocquencourt, France) |  |  |  | HM367524 | HM222860 |
| *Fraxinus platypoda* | A | P00729647 |  | 107 | Pépinière Adeline (La Chapelle Montlinard, France) |  |  |  | HM367513 | HM222855 |
| *Fraxinus potamophila* | A | P00729674 |  | 52 | Arboretum Chèvreloup (Rocquencourt, France) |  |  |  | HM367372 | HM222729 |
| *Fraxinus profunda* | A |  | 2003 | 30 | Arboretum National des Barres (Nogent-sur-Vernisson, France) | HM130648 | GU991705 | HM171508 | HM367529 | HM222865 |
| *Fraxinus profunda* | A |  | 1251-2001 | 254 | Jardin botanique de Montréal (Montréal, Canada) |  |  |  | HM367528 | HM222864 |
| *Fraxinus profunda* | A | P00729582 | 1993.1156*A | 216 | Sir Harold Hillier Garden (Romsey, U.K.) |  |  |  | HM367526 | HM222862 |
| *Fraxinus profunda* | A | P00729593 | OSN93-12-0041-10 | 249 | Botanic Garden Osnabrück (Osnabrück, Deutschland) |  |  |  | HM367527 | HM222863 |
| *Fraxinus profunda* | A |  |  | 125 | Arboretum Bayreuth (Bayreuth, Deutschland) |  |  |  | HM367525 | HM222861 |
| *Fraxinus profunda* | A |  | 2001 | 32 | Arboretum National des Barres (Nogent-sur-Vernisson, France) | HM130655 | GU991714 | | HM367563 | HM222889 |
| *Fraxinus profunda* | A |  | 5525 | 34 | Arboretum National des Barres (Nogent-sur-Vernisson, France) | HM130656 | GU991715 | HM171510 | HM367564 | HM222890 |
| *Fraxinus purpusii* | H | MEXU833610 | 833610 | 18 | Mexico Herbarium (Mexico, Mexico) |  |  |  | HM367530 |  |
| *Fraxinus quadrangulata* | A |  | 518 | 4 | Arboretum National des Barres (Nogent-sur-Vernisson, France) | HM130649 | | HM171514 | HM367532 | HM222867 |
| *Fraxinus quadrangulata* | A |  | 519 | 5 | Arboretum National des Barres (Nogent-sur-Vernisson, France) | HM130650 | GU991706 | HM171523 | | HM222868 |
| *Fraxinus quadrangulata* | A | P00729655 |  | 111 | Pépinière Adeline (La Chapelle Montlinard, France) |  |  |  | HM367531 | HM222866 |
| *Fraxinus raibocarpa* | A |  |  | 126 | Arboretum Bayreuth (Bayreuth, Deutschland) |  |  |  | HM367533 |  |
| *Fraxinus retusa* var. *henryana* | A |  |  | 119 | Pépinière Adeline (La Chapelle Montlinard, France) |  |  |  | HM367534 |  |
| *Fraxinus richardii* | A |  |  | 127 | Arboretum Bayreuth (Bayreuth, Deutschland) |  |  |  | HM367536 | HM222871 |
| *Fraxinus sieboldiana* | W |  | 145 | 166 | Forestry and Forest Products Research Institute (Tsukuba, Japan) |  |  |  | HM367538 |  |
| *Fraxinus sieboldiana* | W |  | 146 | 167 | Forestry and Forest Products Research Institute (Tsukuba, Japan) |  |  |  | HM367539 |  |
| *Fraxinus sieboldiana* | W |  | 147 | 168 | Forestry and Forest Products Research Institute (Tsukuba, Japan) |  |  |  | HM367540 | HM222873 |
| *Fraxinus sieboldiana* | W |  | 148 | 169 | Forestry and Forest Products Research Institute (Tsukuba, Japan) |  |  |  | HM367541 |  |
| *Fraxinus sieboldiana* | W |  | 150 | 170 | Forestry and Forest Products Research Institute (Tsukuba, Japan) |  |  |  | HM367542 |  |
| *Fraxinus sieboldiana* | W |  | 151 | 171 | Forestry and Forest Products Research Institute (Tsukuba, Japan) |  |  |  | HM367543 |  |
| *Fraxinus sieboldiana* | W |  | 152 | 172 | Forestry and Forest Products Research Institute (Tsukuba, Japan) |  |  |  | HM367544 | HM222874 |
| *Fraxinus sieboldiana* | W |  | 153 | 173 | Forestry and Forest Products Research Institute (Tsukuba, Japan) |  |  |  | HM367545 | HM222875 |
| *Fraxinus sieboldiana* | A | P00729599 | 1977.5390*Q | 205 | Sir Harold Hillier Garden (Romsey, U.K.) |  |  |  | HM367546 | HM222876 |
| *Fraxinus sieboldiana* | A |  | 880-1988 | 253 | Jardin botanique de Montréal (Montréal, Canada) |  |  |  | HM367547 | HM222877 |
| *Fraxinus sieboldiana* | A | P00729661 |  | 114 | Pépinière Adeline (La Chapelle Montlinard, France) |  |  |  | HM367537 | HM222872 |
| *Fraxinus sikkimensis* | A |  |  | 120 | Arboretum Frankfürt (Frankfürt, Deutshcland) |  |  |  | HM367548 |  |
| *Fraxinus sogdiana* | A |  | 1002-2003 | 259 | Jardin botanique de Montréal (Montréal, Canada) |  |  |  |  | HM222732 |
| *Fraxinus* sp. | A | P00729573 | 1977.5154 | 235 | Sir Harold Hillier Garden (Romsey, U.K.) |  |  |  | HM367550 | HM222878 |
| *Fraxinus* sp. | A | P00729579 | 1977.5155 | 236 | Sir Harold Hillier Garden (Romsey, U.K.) |  |  |  | HM367551 | HM222879 |
| *Fraxinus* sp. | A |  |  | 71 | Arboretum Chèvreloup (Rocquencourt, France) |  |  |  | HM367553 | HM222881 |
| *Fraxinus* sp. | A |  |  | 70 | Arboretum Chèvreloup (Rocquencourt, France) |  |  |  | HM367552 | HM222880 |
| *Fraxinus* sp. | A | P00729568 |  | 211 | Sir Harold Hillier Garden (Romsey, U.K.) |  |  |  | HM367549 |  |
| *Fraxinus spaethiana* | A | P00729658 | 3825 | 46 | Arboretum National des Barres (Nogent-sur-Vernisson, France) |  | GU991712 | HM171520 | HM367557 | HM222885 |
| *Fraxinus spaethiana* | A | P00729566 | 1977.5400*Q | 208 | Sir Harold Hillier Garden (Romsey, U.K.) |  |  |  | HM367556 | HM222884 |
| *Fraxinus spaethiana* | A | P00729551 | 1977.5614*W | 204 | Sir Harold Hillier Garden (Romsey, U.K.) |  |  |  | HM367555 | HM222883 |
| *Fraxinus spaethiana* | A |  | 1977.5659*R | 203 | Sir Harold Hillier Garden (Romsey, U.K.) |  |  |  | HM367554 | HM222882 |
| *Fraxinus syriaca* | A |  | 2019 | 29 | Arboretum National des Barres (Nogent-sur-Vernisson, France) | HM130653 | GU991710 | HM171506 | HM367376 | HM222734 |
| *Fraxinus syriaca* | A | P00729643 | 2020 | 28 | Arboretum National des Barres (Nogent-sur-Vernisson, France) |  | GU991713 | HM171505 | HM367375 | HM222733 |
| *Fraxinus syriaca* | A |  | 3804 | 39 | Arboretum National des Barres (Nogent-sur-Vernisson, France) | HM130654 | GU991711 | HM171513 | HM367377 | HM222735 |
| *Fraxinus syriaca* | A | P00729665 |  | 116 | Pépinière Adeline (La Chapelle Montlinard, France) |  |  |  |  | HM222736 |
| *Fraxinus syriaca* | A | P00729631 |  | 57 | Arboretum Chèvreloup (Rocquencourt, France) |  |  |  | HM367378 | HM222737 |
| *Fraxinus texensis* | A | P00729589 |  | 267 | Fort Worth Bot. Garden (Fort Worth, U.S.A.) |  |  |  | HM367558 | HM222886 |
| *Fraxinus texensis* | A | P00729597 |  | 269 | Fort Worth Bot. Garden (Fort Worth, U.S.A.) |  |  |  | HM367559 | HM222887 |
| *Fraxinus tomentosa* | A |  | 2298-1961 | 255 | Jardin botanique de Montréal (Montréal, Canada) |  |  |  | HM367562 | HM222888 |
| *Fraxinus tomentosa* | A |  |  | 129 | Arboretum Bayreuth (Bayreuth, Deutschland) |  |  |  | HM367561 |  |
| *Fraxinus tomentosa* | A | P00729669 |  | 118 | Pépinière Adeline (La Chapelle Montlinard, France) |  |  |  | HM367560 |  |
| *Fraxinus trifoliata* | A | P00729611 | 14849*B2 | 242 | Rancho Santa Anna Bot. Garden (Claremont, U.S.A.) |  |  |  |  | HM222891 |
| *Fraxinus trifoliata* | A | P00729613 | 17232*C2 | 241 | Rancho Santa Anna Bot. Garden (Claremont, U.S.A.) |  |  |  | HM367565 |  |
| *Fraxinus turkestanica* | A |  |  | 130 | Arboretum Bayreuth (Bayreuth, Deutschland) |  |  |  | HM367566 | HM222892 |
| *Fraxinus uhdei* | A |  | 19890759 | 247 | Cambridge University Botanic Garden (Cambridge, U.K) |  |  |  | HM367567 | HM222893 |
| *Fraxinus uhdei* | A | P00729634 | 1636 Edgewood Drive | 263 | Canopy Association (Palo Alto, U.S.A.) |  |  |  | HM367569 | HM222895 |
| *Fraxinus uhdei* | A | P00729562 | 1963-0485-P | 265 | Los Angeles County Arboretum and Botanic Garden (Los Angeles, U.S.A.) | |  |  |  | HM222896 |
| *Fraxinus uhdei* | A | P00729558 | 1967-1367-P | 266 | Los Angeles County Arboretum and Botanic Garden (Los Angeles, U.S.A.) | |  |  | HM367570 | HM222897 |
| *Fraxinus uhdei* | A | P00729642 | 3825 Fabian Way | 260 | Canopy Association (Palo Alto, U.S.A.) |  |  |  | HM367568 | HM222894 |
| *Fraxinus uhdei* | A | P00729646 |  | 275 | Colvos Creek Nursery (Seattle, U.S.A.) |  |  |  |  | HM222898 |
| *Fraxinus velutina* | A | P00729684 | 219 | 10 | Arboretum National des Barres (Nogent-sur-Vernisson, France) |  | GU991716 | HM171488 | | HM222899 |
| *Fraxinus velutina* | A |  | 241 | 7 | Arboretum National des Barres (Nogent-sur-Vernisson, France) | HM130659 | GU991720 | HM171525 | HM367572 | HM222903 |
| *Fraxinus velutina* | A | P00729650 | 242 | 6 | Arboretum National des Barres (Nogent-sur-Vernisson, France) | HM130658 | GU991719 | |  | HM222901 |
| *Fraxinus velutina* | A | P00729625 |  | 63 | Arboretum Chèvreloup (Rocquencourt, France) |  |  |  | HM367571 | HM222902 |
| *Fraxinus velutina* | A | P00729663 |  | 115 | Pépinière Adeline (La Chapelle Montlinard, France) |  |  |  |  | HM222900 |
| *Fraxinus velutina* var. *coriacea* | A | P00729550 | 15292*C2 | 240 | Rancho Santa Anna Bot. Garden (Claremont, U.S.A.) |  |  |  | HM367574 | HM222905 |
| *Fraxinus velutina* var. *coriacea* | A | P00729687 | 5942*B3 | 239 | Rancho Santa Anna Bot. Garden (Claremont, U.S.A.) |  |  |  | HM367573 | HM222904 |
| *Fraxinus velutina* var. *coriacea* | A | P00729623 |  | 64 | Arboretum Chèvreloup (Rocquencourt, France) |  |  |  | HM367575 | HM222906 |
| *Fraxinus velutina* var. *glabra* | A | P00729654 | 1149 | 36 | Arboretum National des Barres (Nogent-sur-Vernisson, France) |  | GU991717 | HM171511 | HM367576 | HM222907 |
| *Fraxinus velutina* var. *glabra* | A |  | 1150 | 37 | Arboretum National des Barres (Nogent-sur-Vernisson, France) | HM130657 | GU991718 | HM171512 | HM367577 | HM222908 |
| *Fraxinus velutina* var. *toumeyi* | A | P00729563 | 1977.0656*U | 227 | Sir Harold Hillier Garden (Romsey, U.K.) |  |  |  | HM367579 | HM222910 |
| *Fraxinus velutina* var. *toumeyi* | A | P00729549 | 1977.5304*W | 206 | Sir Harold Hillier Garden (Romsey, U.K.) |  |  |  | HM367578 | HM222909 |
| *Fraxinus xanthoxyloides* | A | P00729580 | 1986.2709*A | 220 | Sir Harold Hillier Garden (Romsey, U.K.) |  |  |  | HM367581 | HM222912 |
| *Fraxinus xanthoxyloides* | A |  |  | 62 | Arboretum Chèvreloup (Rocquencourt, France) |  |  |  | HM367582 | HM222913 |
| *Fraxinus xanthoxyloides* | A | P00729659 |  | 113 | Pépinière Adeline (La Chapelle Montlinard, France) |  |  |  | HM367580 | HM222911 |
| *Fraxinus xanthoxyloides* var. *dimorpha* | A | P00729694 | 229 | 8 | Arboretum National des Barres (Nogent-sur-Vernisson, France) |  | GU991721 | HM171526 | HM367584 | HM222915 |
| *Fraxinus xanthoxyloides* var. *dimorpha* | A |  | 259 | 9 | Arboretum National des Barres (Nogent-sur-Vernisson, France) | HM130660 | |  |  | HM222916 |
| *Fraxinus xanthoxyloides* var. *dimorpha* | A | P00729676 | 3018 | 51 | Arboretum National des Barres (Nogent-sur-Vernisson, France) |  |  |  | HM367583 | HM222914 |
| *Fraxinus xanthoxyloides* var. *dumosa* | A | P00729553 | 1978.1290*Q | 200 | Sir Harold Hillier Garden (Romsey, U.K.) |  |  |  | HM367585 |  |
| *Fraxinus xanthoxyloides* var. *dumosa* | A | P00729673 |  | 67 | Arboretum Chèvreloup (Rocquencourt, France) |  |  |  | HM367586 | HM222917 |
| ***Outgroup*** |  |  |  |  |  |  |  |  |  |  |
| *Jasminum nudiflorum* |  |  | NC008407 |  | GenBank | JNC0181 | JNC0253 | JNC0024 | JNC0000- JNC0005 | JNC1250- JNC1260 |
